# American Medical Association

**Published:** 1855-06

**Authors:** 


*American Medical Association.*

We publish in the present number of the Journal a very full report of the proceedings of the Association at its recent annual meeting in Philadelphia. Our readers will perceive that in addition to the daily record of proceedings, we have included in our report the annual address of the President, and the and the address of Dr. Hays with the reply of Mayor Conrad, on the occasion of the reception of the members of the Association in *Independence Hall.* These have necessarily crowded out of this number much other matter that might have been deemed by some as of more *practical* value. But if we mistake not, these addresses will be read with much pleasure by all our subscribers. That of Mayor Conrad, particularly, is one of the most eloquent and appropriate addresses to which we have ever listened on any similar occasion. The recent meeting of the Association in Philadelphia, was in all respects one of the best ever enjoyed by its members. The number of members in attendance was very large, amounting to near 550, and representing very fully every section of the country except, perhaps, the extreme south-west. The reports and papers presented and read were as numerous and interesting as at any former meeting; and the proceedings throughout were conducted with the most perfect order and good feeling.

At no period since the day of its organization has the Association presented so good a prospect for a long career of prosperity and usefulness, as at the present time.

